# Avoidance and early diagnosis of erroneous permanent pacemaker placement into the left ventricle: A case report

**DOI:** 10.1002/ccr3.4508

**Published:** 2021-07-21

**Authors:** Abdelrahman Osman, Ali Ahmad

**Affiliations:** ^1^ College of Arts and Sciences The Ohio State University Columbus OH USA; ^2^ Department of Cardiovascular Imaging Mercy Tiffin Cardiovascular Center Tiffin OH USA

**Keywords:** cardiovascular disorders

## Abstract

Diagnosis of a pacemaker inserted through the subclavian artery is essential due to risk of thromboembolism. Fluoroscopy, ECG, and other imaging techniques must be used to diagnose the condition, and appropriate treatment path must be administered.

## Graphical abstract

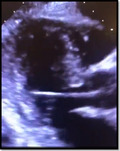

## 1 INTRODUCTION

Implantation of pacemakers has increased in recent decades and has become an important tool to treat bradycardia. With access commonly through the left subclavian or cephalic vein, pacemaker leads are positioned into alternative pacing sites where they can stimulate the heart to restore normal rhythm.^1^ Pacemaker lead insertion through the left subclavian artery via the aortic valve into the left ventricular cavity is an extremely rare iatrogenic complication. The frequency of this complication is unknown but believed to be largely unreported. Common complications resulting from entry through the subclavian artery include thromboembolic events.^2^

Because of these complications, immediate identification and treatment are required before the patient leaves the electrophysiology laboratory, usually by using fluoroscopy, pacemaker interrogation, or a 12‐lead electrocardiogram (ECG). If left undiagnosed, improperly placed leads can lead to serious systemic thromboembolism. Surgical extraction of an arterial lead can also lead to major life‐threatening vascular complication.^3^ We report a case of a 59‐year‐old man who inadvertently had a pacemaker lead implanted via the subclavian artery.

## 2 CASE REPORT

A 59‐year‐old man with a history of chronic obstructive pulmonary disease presented to the emergency room with severe sinus bradycardia and hypotension. Due to his history of recurrent dizziness and presyncope, prior work up with Holter cardiac event monitor showed predominant sinus bradycardia with no evidence of chronic incompetence and no clear correlation of his symptoms to the bradycardia episodes. His most recent echocardiogram showed normal left ventricular systolic function and wall motion. Myocardial perfusion study done for recurrent chest pain showed normal myocardial perfusion and normal ejection fraction on Gated SPECT. Tilt table test was markedly abnormal consistent with severe dysautonomia. His baseline blood pressure was 113/70 mmHg, and his HR was 49 beats per minute. Only two minutes into the test, his blood pressure dropped to 60/29 mmHg while his heart rate remained at 47 bpm. The patient was offered pacemaker therapy after his abnormal tilt table test but he refused.

A few weeks later, the patient presented to the emergency room with syncope. Initial ECG showed severe sinus bradycardia, heart rate in the low 40 s with normal PR interval, and left anterior fascicular block. He was hypotensive and diaphoretic. He denied any chest pain, pressure, or tightness. There were no significant signs of cerebral hypoperfusion. The patient was not on any bradycardia‐inducing medications, and initial blood work showed no reversible cause of his severe sinus bradycardia.

The patient was started on intravenous fluid hydration, and a bedside echocardiogram was obtained. Echocardiogram showed normal biventricular systolic function and no significant valvular abnormalities. Atropine was given but resulted in minimal, transient improvement in his heart rate to the low 50 s. The systolic blood pressure remained below 80 mmHg. Dopamine followed by norepinephrine drips were administered, and the patient was sent to the electrophysiology laboratory for dual‐chamber permanent pacemaker therapy.

**FIGURE 1:**
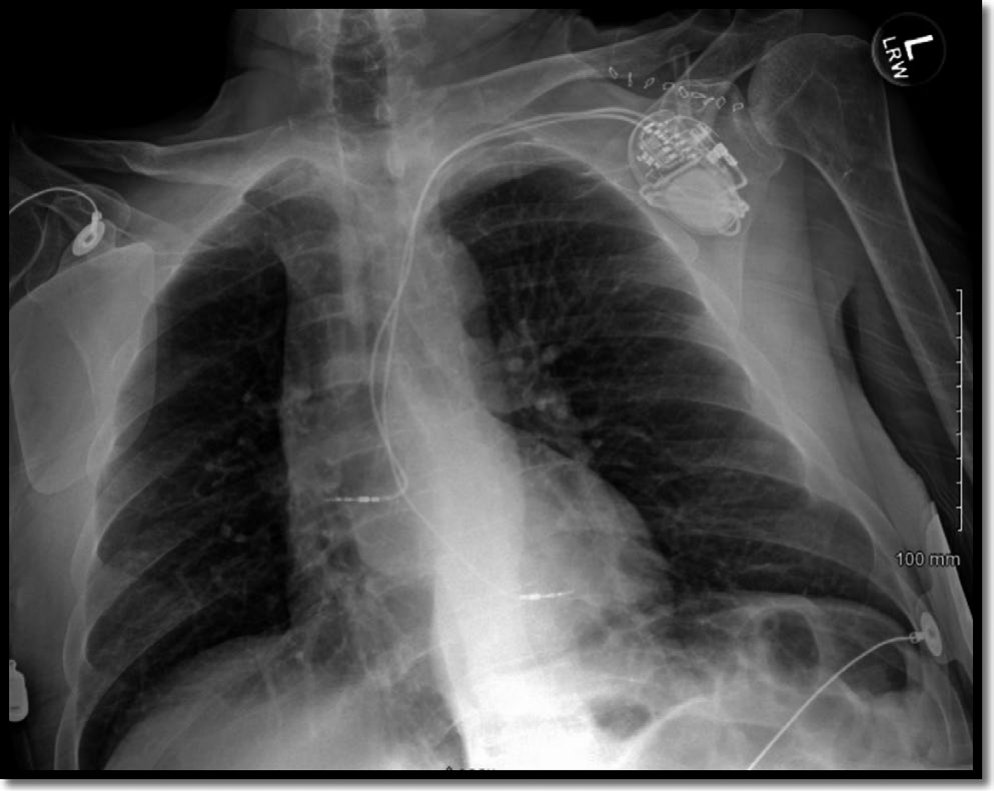
Anteroposterior view of the chest showing no evidence of pneumothorax with the atrial lead in good position while the ventricular lead is higher. This can suggest inadvertent lead placement but lateral chest X‐ray is needed for further evaluation

**FIGURE 2:**
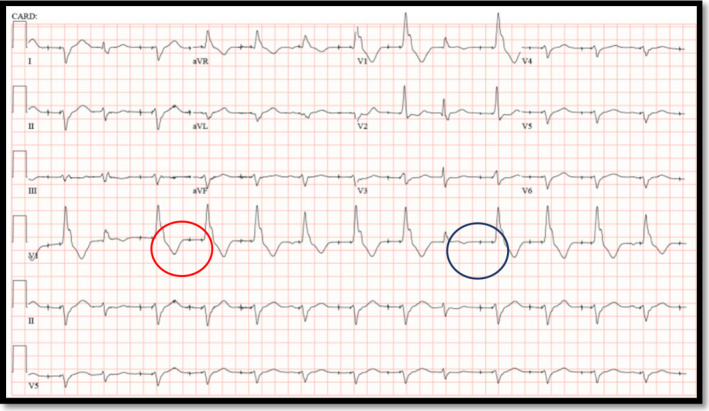
A 12‐lead ECG showing atrial pacing with long AV delay (blue circle) and intermittent ventricular pacing (red circle). The key point is that the ventricular paced beats are conducted with right bundle branch block morphology rather than the expected left bundle branch block with right ventricular pacing

**FIGURE 3:**
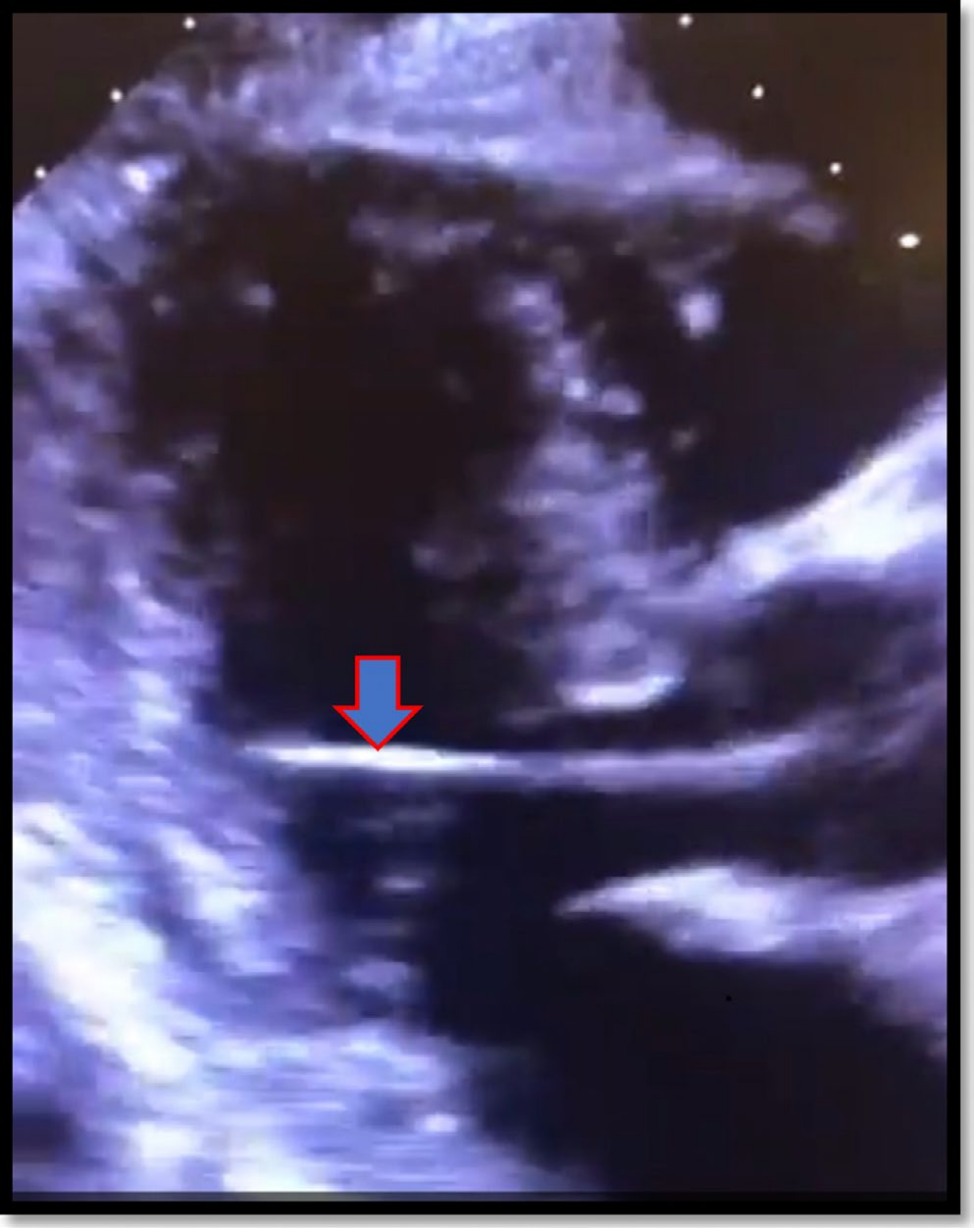
Parasternal long‐axis view showing pacemaker lead (arrow) crossing the aortic valve into the left ventricular cavity and fixed to the inferolateral wall of the left ventricle

**FIGURE 4:**
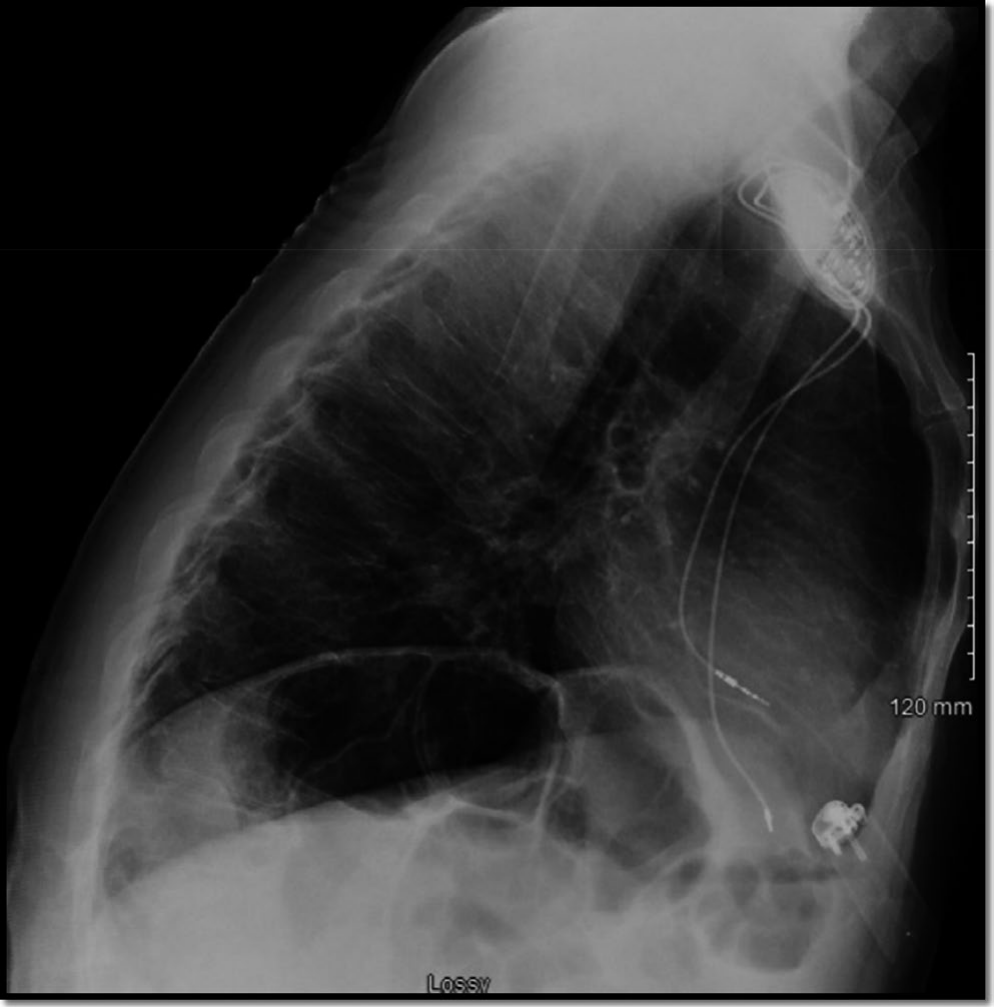
Lateral chest X‐ray view post‐pacemaker revision with the ventricular lead directed anteriorly suggesting right ventricular position

During implantation of a permanent pacemaker, the right atrial lead was appropriately placed via the left subclavian vein into the right atrial appendage. However, the ventricular lead was placed via the subclavian artery into the left ventricular cavity. He was admitted to the hospital overnight and a chest X‐ray was obtained the next day, showing no evidence of pneumothorax (Figure 1). Surprisingly, his ECG showed atrial pacing with long AV delay and intermittent ventricular pacing with the paced ventricular beats showing right bundle branch block morphology contrary to the expected left bundle branch block morphology seen when the lead is inside the right ventricular cavity (Figure 2). A limited bedside Echo was obtained that showed the ventricular lead clearly crossing the aortic valve to the left ventricular cavity and fixed into the inferolateral wall of the left ventricle (Figure 3). Early recognition of this serious pacemaker implantation complication led to pacemaker revision the same day with extraction of the arterial lead and reinsertion of the ventricular lead via the left subclavian vein into the right ventricular cavity (Figure 4). The pacemaker revision went uncomplicated.

## 3 DISCUSSION

Cardiac pacemakers and defibrillators are cornerstones in the management of patients with heart rate and/or rhythm problems. There is a steady growth in the use of pacemaker and defibrillator therapy in the United States and across the world.^1^, ^2^, ^3^ Although commonly inserted with access through the left subclavian or cephalic vein, inadvertent entry through the subclavian artery can lead to iatrogenic complications, including thromboembolism. Quick diagnosis of this event is essential, as leaving the lead is associated with an increased risk of life‐threatening thromboembolism. Thrombus formation on the lead can occur within days, and fibrous tissue may begin developing in just a few months.^4^

There are several methods available to avoid the occurrence of such events. (1) Cephalic vein access or an echo‐guided puncture of the extra‐thoracic subclavian vein should be the first choice of vascular access. (2) On obtaining vascular access, careful observation of the color of pulsatility of the returning blood should be practiced to determine whether it is from the artery or vein. (3) If subclavian artery puncture is suspected, arteriography may be used to identify the accessed vessel. In addition, successful venipuncture may be observed as the guidewire advances into the inferior vena cava under fluoroscopy. If there are ventricular premature beats with right bundle branch block pattern soon after the guidewire enters the ventricular cavity, the possibility of the guidewire entering through the artery must be considered. (4) After the pacemaker is implanted, correct positioning of the pacemaker may be confirmed by fluoroscopy in post‐anterior view, 45‐degree left anterior oblique view and 30‐degree right anterior oblique view. (5) Post‐pacemaker ECG interpretation is very important particularly if ventricular pacing is present. Right ventricular pacing causes LBBB morphology, whereas left ventricular pacing should be suspected if ECG shows RBBB morphology with tall R‐waves in lead in V1.^5^, ^6^ (6) If ventricular pacing is absent at baseline as in patients with sick sinus syndrome or intermittent heart block then performing ECG during threshold testing to identify the QRS morphology should be considered. (7) Post‐pacemaker chest X‐ray should be obtained in anteroposterior and lateral views whenever possible.

Removal of the lead is one solution to avoid thromboembolic events but comes with risk for other complications. Extraction of transarterially placed leads is associated with high thromboembolic risk and risk of bleeding from the arterial entry site. Furthermore, lead removal can lead to ventricular perforation or cardiac tamponade and may cause trauma to the aorta, aortic valve, and coronary arteries.^7^ Given the direct connection to systemic circulation, any lead manipulation can potentially lead to systemic embolization. Due to the unnecessary risks pertaining to surgical removal, conservative management with lifelong anticoagulation may be an acceptable alternative in chronically implanted left ventricular cavity leads.^8^, ^9^

## 4 CONCLUSION

Insertion of a pacemaker lead through the subclavian artery is extremely rare and believed to be markedly underreported. Adverse events resulting from this iatrogenic complication include arterial injury and thromboembolic events. Due to the severity of these risks, immediate identification and diagnosis of this complication are essential. Careful interpretation of post‐implantation ECG lead is of extreme value to identify this complication. Proper utilization of imaging modalities including lateral chest X‐ray view, echocardiography, and fluoroscopy should be adopted whenever improper lead placement is suspected.

## CONFLICT OF INTEREST

The authors declare no conflict of interest.

## AUTHOR CONTRIBUTIONS

AA: analyzed and studied patient complaints, images, and eventual diagnosis, as well as reviewed all drafts of article. AO: wrote main manuscript and critically reviewed all drafts.

## ETHICAL APPROVAL

Written with consent of patient.

## CONSENT STATEMENT

Published with written consent of the patient.

## Data Availability

Data sharing is not applicable as no new data sets were generated or analyzed.
